# Jonas Salk (1914-1995): Pioneering the Fight Against Polio and Beyond

**DOI:** 10.7759/cureus.69681

**Published:** 2024-09-18

**Authors:** Hemlata Sahu, Sonali G Choudhari, Abhay Gaidhane, Swarupa Chakole

**Affiliations:** 1 Department of Community Medicine, Datta Meghe Institute of Higher Education andResearch, Jawaharlal Nehru Medical College, School of Epidemiology and Public Health, Wardha, IND; 2 Department of Community Medicine, Datta Meghe Institute of Higher Education and Research, Jawaharlal Nehru Medical College, School of Epidemiology and Public Health, Wardha, IND; 3 Department of Community Medicine, Jawaharlal Nehru Medical College, School of Epidemiology and Public Health, Datta Meghe Institute of Higher Education & Research, Wardha, IND

**Keywords:** clinical trial, historical vignette, medical innovation, polio vaccine, public health

## Abstract

Jonas Salk (October 28, 1914 - June 23, 1995) was an American medical researcher celebrated for his pioneering work in virology, particularly the development of the first successful polio vaccine. This review highlights Salk's multifaceted talent and contributions. His research on the poliovirus led to the creation of the inactivated polio vaccine, proving that it could prevent the disease. In 1955, the discovery of the polio vaccine was a pivotal moment in the fight against poliomyelitis. Salk's contributions are celebrated in the record of medical history, highlighting his impact on modern medicine and public health. As a professor of bacteriology, preventive medicine, and experimental medicine, Salk's scientific journey, from his innovative methods to the creation and widespread use of the inactivated polio vaccine, helped eradicate polio from various parts of the world. His contributions beyond polio, such as his work on the influenza vaccine and the founding of the Salk Institute for Biological Studies, are also well known. By exploring Salk's legacy, this review examines how his work and dedication continue to influence modern medicine, public health, and science, impacting humanity.

## Introduction and background

**Figure 1:**
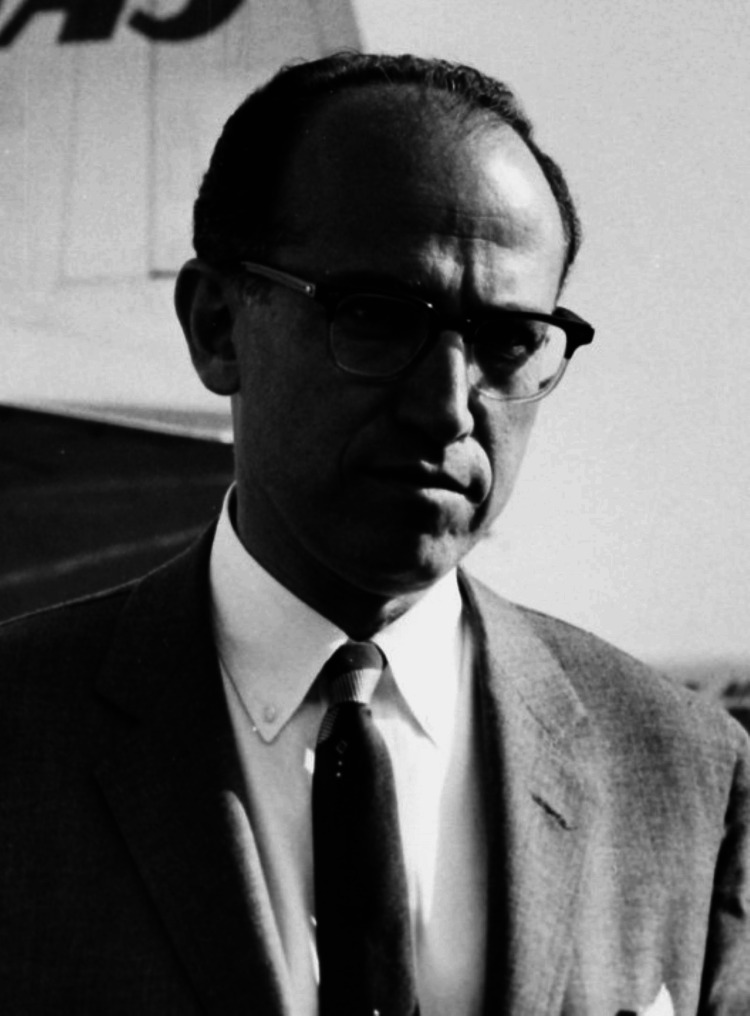
Dr Jonas Edward Salk Credit: SAS Scandinavian Airlines Source: [1]

Jonas Salk (Figure 1), an American virologist and medical researcher, developed one of the first effective vaccines against polio. The primary aim of this article is to explore the significant contributions of Jonas Salk to the development of the polio vaccine, which has saved numerous lives worldwide by preventing the disease and reducing its impact on public health. Beyond polio, Salk's contributions extended to other research fields, highlighting his relentless pursuit of scientific advancement and humanitarian efforts.

## Review

Jonas Salk’s life and career

Jonas Salk was born on October 28, 1914, in New York City, USA, to a family of Polish-Jewish immigrants who followed the Orthodox faith [2]. He was the first child born to his parents, who emphasized the importance of education despite their lack of formal schooling. Salk received continuous academic support throughout his youth, which enabled him to complete high school at the age of 15. Then, he attended the City College of New York, initially planning to study the law. However, his interest shifted to medical science, and in 1934, he graduated with a Bachelor of Science degree [2].

Salk went on to pursue a career in medical research at New York University, where he entered medical school at the age of 19. He earned his M.D. in 1939, and during his time there, he collaborated with Thomas Francis Jr., focusing on killed-virus immunology studies. This collaboration continued during Salk's fellowship at the University of Michigan, where they conducted groundbreaking research on the influenza virus [3]. During World War II, the US Army-funded their work, leading to the creation of a flu vaccine in 1943. This vaccine, made from a dead virus strain, produced antibodies without causing illness, marking a significant advancement in vaccine development [4].

In 1947, Salk was appointed as an associate professor of bacteriology and the director of the Virus Research Laboratory at the University of Pittsburgh School of Medicine [2]. His pioneering work with Francis on the flu vaccine laid the groundwork for his later polio vaccine development. This achievement represented a notable milestone in medical research and had a lasting impact on vaccine production [3,5].

Discovery of a vaccine against poliomyelitis

Jonas Salk is recognized for developing the first successful vaccine for poliomyelitis (infantile paralysis). Poliomyelitis outbreaks had worsened, with approximately 58,000 cases and more than 3000 deaths were recorded in the United States in 1952 [6]. Doctors Thomas Francis Jr. and Jonas Salk led the study which later became closely linked to the polio vaccine. Salk developed the first successful polio vaccine between 1952 and 1955, and testing began [6]. In 1954, Salk conducted trials on himself and his own family, then proceeded to carry out wide-ranging trials on more than 1.3 million children [6]. After the vaccine became available in the United States on April 12, 1955, the number of polio cases dropped significantly, and by 1995, polio had been eradicated throughout the entire Western Hemisphere [6,7]. In 1945, the initial influenza vaccine was authorized for military usage, and a year later, in 1946, it received approval for civilian use [6]. 

Challenges in polio research work

Salk discovered that factors beyond control, such as social, political, and economic issues, were the primary obstacles in efforts to eradicate polio. He could not identify any significant factors that directly contributed to the eradication of polio [8].

World Polio Day (October 24)

World Polio Day, observed on October 24, was established by Rotary International to honor Jonas Salk, who led the team that developed first polio vaccine. The introduction of Salk's inactivated poliovirus vaccine, followed by the widespread distribution of the oral poliovirus vaccine developed by Albert Sabin, led to the launch of the Global Polio Eradication Initiative in 1988. Since then, this initiative has reduced global polio cases by 99% [9].

Salk's work beyond the polio vaccine

Even though Salk created the first polio vaccine, he did not receive the Nobel Prize or joined the National Academy of Sciences. Despite his efforts to avoid attention, he was still criticized for seeking public praise due to his pioneering work. Jonas Salk's contributions to science and medicine extended far beyond the development of the polio vaccine. His work spanned various research fields and humanitarian initiatives, showcasing his unwavering dedication to enhancing public health and advancing scientific understanding [10]. AIDS vaccine research, cancer research, multiple sclerosis research, public health and policy, philosophical and ethical contributions, and the Salk Institute for Biological Studies are some of Salk's key achievements beyond polio vaccine. His continued work in various areas of medicine and his dedication to using science for the greater good have left a lasting mark on his legacy. His efforts continue to inspire scientists and public health workers even today [11].

AIDS vaccine work

Jonas Salk was dedicated to creating vaccines and worked hard to make them successful. Later in his life, he also worked on a vaccine to help people with HIV prevent developing AIDS. He stated that using inactivated viruses could help the immune system fight infections. His work had made significant progress, and it is important to honour his legacy and support those affected by diseases [12]. Starting in the mid-1980s, Salk conducted studies aimed at creating an AIDS vaccine. In collaboration with Kevin Kimberlin, Jonas Salk co-founded the Immune Response Corporation, where they developed an immunologic therapy called Remune. Despite their efforts, the product faced challenges in securing insurance coverage, leading to the project's discontinuation in 2007, twelve years after Salk's passing [13].

Books by Jonas Salk

Dr. Jonas Salk was an enthusiastic writer who published many books and a lot of research articles (Table 1).

**Table 1:** Books by Dr. Jonas Salk

Category	Title	Year
Demography	World population and human values: a new reality [14]	1981
Philosophy	The survival of the wisest [15]	1973
Science	Man unfolding [16]	1972
Philosophy	Anatomy of reality: merging of intuition and reason [17]	1983
Science	Infectious molecules and human disease [18]	1962
Political science	A new reality: human evolution for sustainable future [19]	2018
Human biology, social human & behavior	How like an angel: biology and the nature of man [20]	1975

Awards and recognition

Dr. Jonas Salk received many awards and honors for his revolutionary work on the polio vaccine and his contributions to medical science (Table 2).

**Table 2:** Some of the notable awards and prizes by Salk.

Awards	Year
Albert Lasker Award [21]	1956
Presidential Medal of Freedom [22]	1977
The Jawaharlal Nehru Award [23]	1975
A Congressional Gold Medal [24]	1956
The Academy of Achievement's Golden Plate Award [5]	1976
The James D. Bruce Memorial Award [25]	1958
Humanist of the Year Award [26]	1976

Legacy

Dr. Salk spent his last years working on an AIDS vaccine. He passed away on June 23, 1995, at the age of 80, leaving behind an incredible scientific legacy. In 2014, people around the world celebrated the 100th anniversary of his birth, honouring his significant contributions to humanity. His wife, Françoise Gilot, was a talented and famous painter who created art for over 50 years. She lived in New York City for many years and passed away on June 6, 2023, at the age of 101 [5].

## Conclusions

This review emphasizes on Jonas Salk's innovative contributions that revolutionized our understanding and control of poliomyelitis, significantly impacting public health approaches to the disease and saving countless lives. Salk's development of the first effective polio vaccine laid the groundwork for future research and vaccination initiatives. Despite facing considerable challenges, Salk's unwavering dedication to scientific inquiry ensured his lasting legacy. Beyond his monumental scientific achievements, Salk was a visionary who established the Salk Institute for Biological Studies, fostering continued advancements in research. His multifaceted legacy highlights his contributions to medicine and the broader scientific community, underscoring the profound and lasting impact of his work on global health.
